# Effect of convalescent plasma transfusion on outcomes of coronavirus disease 2019: a meta-analysis with trial sequential analysis

**DOI:** 10.1007/s00540-023-03171-x

**Published:** 2023-02-22

**Authors:** Sameh M. Hakim, Ghosoun M. A. Chikhouni, Mona A. Ammar, Akram M. Amer

**Affiliations:** Department of Anesthesiology, Intensive Care and Pain Management, Faculty of Medicine, Ain Shams University, 15 Gamal Noah Street, Almaza, Heliopolis, Cairo, 11341 Egypt

**Keywords:** Convalescent plasma, Transfusion, CP, CPT, Coronavirus disease 2019, COVID-19, Novel coronavirus, SARS-COV-2, Severe acute respiratory syndrome coronavirus 2

## Abstract

The aim of this review was to update evidence for benefit of convalescent plasma transfusion (CPT) in patients with coronavirus disease 2019 (COVID-19). Databases were searched for randomized controlled trials (RCT) comparing CPT plus standard treatment versus standard treatment only in adults with COVID-19. Primary outcome measures were mortality and need for invasive mechanical ventilation (IMV). Twenty-Six RCT involving 19,816 patients were included in meta-analysis for mortality. Quantitative synthesis showed no statistically significant benefit of adding CPT to standard treatment (RR = 0.97, 95% CI = 0.92 to 1.02) with unimportant heterogeneity (Q(25) = 26.48, *p* = .38, *I*^*2*^ = 0.00%). Trim-and-fill-adjusted effect size was unimportantly changed and level of evidence was graded as high. Trial sequential analysis (TSA) indicated information size was adequate and CPT was futile. Seventeen trials involving 16,083 patients were included in meta-analysis for need of IMV. There was no statistically significant effect of CPT (RR = 1.02, 95% CI = 0.95 to 1.10) with unimportant heterogeneity (Q(16) = 9.43, *p* = .89, *I*^*2*^ = 3.30%). Trim-and-fill-adjusted effect size was trivially changed and level of evidence was graded as high. TSA showed information size was adequate and indicated futility of CPT. It is concluded with high level of certainty that CPT added to standard treatment of COVID-19 is not associated with reduced mortality or need of IMV compared with standard treatment alone. In view of these findings, further trials on efficacy of CPT in COVID-19 patients are probably not needed.

**Supplementary Information:**

The online version contains supplementary material available at 10.1007/s00540-023-03171-x.

## Introduction

As of 18 December 2022, the severe acute respiratory syndrome coronavirus 2 (SARS-CoV-2) has been reported to be responsible for approximately 650 million confirmed cases and more than 6.6 million deaths worldwide [1]. Despite optimistic expectations, specific therapeutic options are still limited and efforts at mass vaccination have fallen short of offering long-lasting immunity or providing effective coverage of the entire population, especially in underdeveloped countries [2, 3]. Disappointingly, latest data from the World Health Organization (WHO) indicate that over 3.7 million new cases are still being reported and over 10,000 victims succumb to COVID-19 each week. Besides, there is good evidence that currently reported incidence rates do underestimate the actual incidence owing to the progressive relaxation in COVID-19 testing strategies adopted worldwide resulting in fewer tests being performed and subsequently fewer cases being detected [1].

Transfusion of convalescent plasma (CP) obtained from donors who recovered from a recent SARS-CoV-2 infection has been suggested as a treatment option for coronavirus disease 2019 (COVID-19). Providing passive immunity against the virus via transfusion of high titers of neutralizing antibodies contained in CP is the rationale underlying its use in this context [4].

Convalescent plasma transfusion (CPT) has been previously employed to combat similar viral outbreaks with promising results. Evidence from these trials suggests that CPT could be more effective in patients with high viral load and in those with more progressive or more severe forms of the disease. Moreover, it has been suggested that early administration of CP could improve outcome under such circumstances when neutralizing antibodies have not been produced in adequate amount by the host and the viral load is yet high [5]. On the other hand, the risk of transfusion-related reactions does not seem to be significantly higher in this setting than that associated with plasma transfusion for other indications [3]. Consequently, the concept of utilizing CPT has been extended to the current SARS-CoV-2 problem but with inconsistent results, even at the highest level of evidence as obtained from meta-analyses [6–8].

The present meta-analysis updates the results of an antecedent meta-analysis published over 1 year ago [9]. As more evidence is being added from newly appearing publications and in absence of convincing indications that the problem of COVID-19 has vanished, we deemed it pertinent to update our evidence on the benefit or futility of CPT in management of COVID-19. Our prime aim was to re-examine the effect of using CPT on mortality and need of invasive mechanical ventilation (IMV) in patients with COVID-19 as seen in the light of currently accumulating evidence.

## Methods

### Eligibility criteria

This systematic review and meta-analysis was conducted in concord with the Preferred Reporting Items for Systematic Reviews and Meta-Analyses (PRISMA) guidelines [10]. The study protocol defining inclusion criteria for studies, search methodology and statistical analysis was defined a priori. The protocol obtained approval of the Research Ethics Committee of Ain Shams University Faculty of Medicine (FWA 000,017,585) and was registered at the local institutional registry under number FMASU 151/2021, dated March 21, 2021.

Randomized clinical trials (RCT) comparing CPT added to standard treatment versus standard treatment only in polymerase chain reaction (PCR)-confirmed adult patients (> 18 years of age) were eligible. The review was limited to articles published in English language, including pilot studies and preprints, without restrictions to the date of publication. Non-original studies, studies not providing data regarding the outcome measures of interest or studies conducted on animals were not eligible.

### Search strategy

Database search was commenced on April 3, 2021 and was updated on three-monthly basis thereafter. The last update was carried out on September 11, 2022 prior to publication. Two authors (AMA and GMAC) independently performed electronic search in the Medline/Pubmed, Web of Science, EMBASE, Cochrane Database of systematic reviews (CDSR), Wiley Online Library, and Scopus databases. Initially, the presence of controlled descriptors (such as MeSH terms and Emtree) and their synonyms (key words) was identified in each database. The search terms were combined using the operators ‘OR’ and ‘AND’. Then, a search strategy incorporating MeSH terms and free-text words, such as (“convalescent plasma” OR “convalescent plasma transfusion” OR “convalescent plasma therapy”) AND (“COVID-19” OR “novel corona virus” OR “SARS-CoV-2” OR “severe acute respiratory syndrome coronavirus 2”) was used. In order to identify randomized clinical trials, we applied the term: AND (“randomized controlled trial” OR “RCT”). Search was limited to titles published in English. No restriction was placed on date or status of publication. The references of all eligible studies were reviewed to identify other potentially eligible studies. Both authors independently screened the search results by title and abstract. Studies selected at this level were further assessed for eligibility by examination of full text. Disagreements were resolved by seeking opinion of the first author (SMH).

### Outcome measures

The primary outcome measures were mortality and need for invasive mechanical ventilation (IMV). Owing to the wide variability among trials regarding the extent of follow up for mortality, we limited our analysis to events occurring within 90 days from inclusion. The secondary outcome measure was the incidence of transfusion-related adverse events (AE) such as transfusion-related acute lung injury (TRALI), volume overload, or anaphylaxis.

### Data extraction

Data were extracted and fed into a spreadsheet by two independent authors (AMA, GMAC) and then were reviewed by the first author (SMH). The following information was extracted: authors’ names, year of publication, country where the study was conducted, study design, number and demographic characteristics of participants, disease severity at inclusion, timing and duration of CP administration, dosages of CP, concomitant therapy, treatment outcomes and conclusions of authors. Data required for quantitative synthesis of each outcome measure of interest was then tabulated in a spread sheet. This included study identifier, total number of patients assigned to either study arm, and number of events recorded in either arm.

### Risk of bias assessment

Two researchers (AMA, GMAC) assessed methodological bias in each selected study independently. Results were compared by a third researcher (MAA) and disagreements were discussed with the first author (SMH) to resolve any discrepancies. The Cochrane Risk-of-Bias Tool for Randomized Trials Version 2.0 (RoB 2) was used to assess quality of randomized clinical trials (RCT) [11].

### Statistical analysis

Statistical analysis was done using the Stata© software version 16.1 (StataCorp LLC, 4905 Lakeway Drive, College Station, TX 77,845, USA) and Trial Sequential Analysis Software (TSA) version 0.9.5.5 Beta (Copenhagen Trial Unit, Copenhagen, Denmark, 2011).

Binary outcomes are expressed as risk ratio (RR) and 95% confidence interval (95% CI). Heterogeneity across studies was tested using the Cochran Q chi-squared test and the I-squared statistic (*I*^*2*^). A *p* value < 0.1 for the Cochran Q test and/or an *I*^*2*^ > 50% is regarded as evidence of significant heterogeneity. Pooling of estimates was done using a restricted maximum likelihood (REML) random effects model. Leave-one-out meta-analysis was conducted to identify influential studies and assess the robustness of the analysis. Publication bias was examined using funnel plot of the log RR versus the standard error of the RR together with Egger’s and Begg’s tests for funnel plot asymmetry [12, 13]. The Duval and Tweedie trim-and-fill method was employed to impute missing studies, if any, and to adjust the point estimate accordingly [14]. The certainty of evidence for either outcome measure was assessed using the GRADEpro system [15]. For trial sequential analysis (TSA), we targeted a relative risk reduction (RRR) of 20% as a clinically meaningful effect size and set the final type 1 error at 0.05 and type 2 error at 0.2. Based on observed event rates in control arm, we assumed event rates of 22% and 15% for mortality and need of IMV, respectively.

## Results

**Fig. 1:**
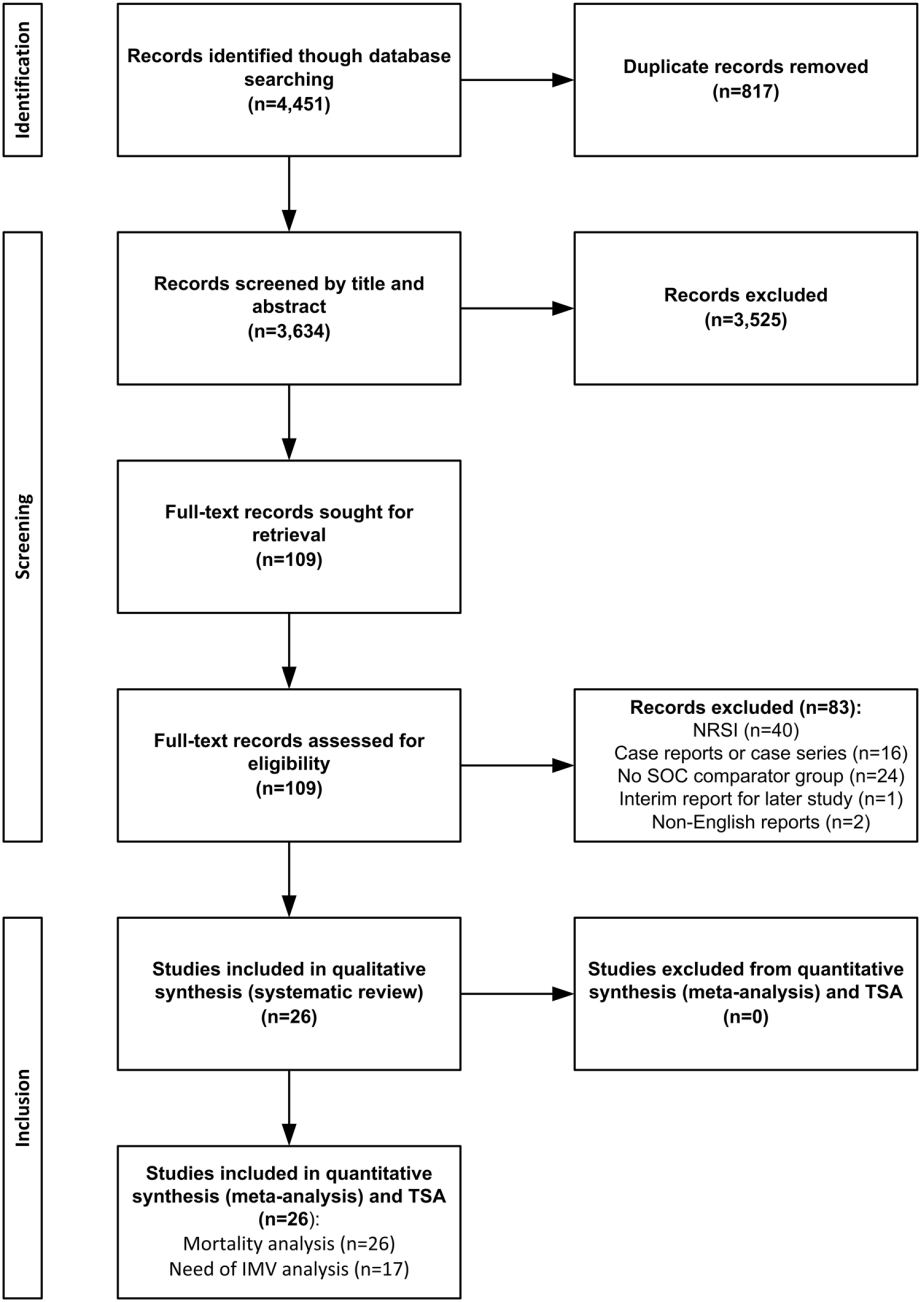
Preferred Reporting Items for Systematic Reviews and Meta-Analyses (PRISMA) flow chart illustrating various stages of the review including search of databases, identification and screening of citations, inclusion and exclusion of trials, qualitative and finally quantitative synthesis of data. *NRSI*, non-randomized studies of intervention, *SOC* standard of care

Searching the literature returned 4,451 relevant titles. After exclusion of duplicate records (*n* = 817), 3,634 reports were examined by title and abstract, out of which 3,525 titles were excluded for ineligibility. The remaining 109 records were examined by full text. Eighty-Three full-text articles were excluded because they were non-randomized studies of intervention (NRSI) (*n* = 40), case reports or case series (*n* = 16), single-arm clinical studies with no comparator group receiving standard of care only (*n* = 24), interim analysis for a study published later (*n* = 1) or were published in languages other than English with only the abstract provided in the English language (*n* = 2). Twenty-Six RCT including 19,816 patients were eligible. All 26 trials provided valid information on mortality that was utilized for quantitative synthesis (meta-analysis) regarding this outcome measure [16–41]. Endpoint of mortality varied among studies. Thirteen trials examined 28-day mortality [16–21, 26, 29, 32, 33, 37, 38, 40], six trials examined 30-day mortality [22, 23, 31, 36, 39, 41], three trials examined 60-day mortality [25, 28, 34] and a single trial examined 90-day mortality [24]. Three trials broadly reported in-hospital overall mortality [27, 30, 35]. As regards need of IMV, 17 trials (16,083 patients) provided valid information for quantitative synthesis. Search results are illustrated in Fig. 1 and characteristics of included studies are shown in Online Resource 1. Risk of bias assessment for individual trials is shown in Online Resource 2 and overall risk of bias in included trials is shown in Online Resource 3. The majority of included studies (22/26) were qualified as high-quality [16–19, 26, 28–34, 37–41], fulfilling all five criteria of the RoB 2 Tool [11]. One or more criteria of the RoB 2 Tool [11] were either unclearly reported or completely missing in four studies [20, 27, 35, 36], which were regarded as low-quality.

### Mortality

#### Pooling of estimates and assessment of heterogeneity

**Fig. 2:**
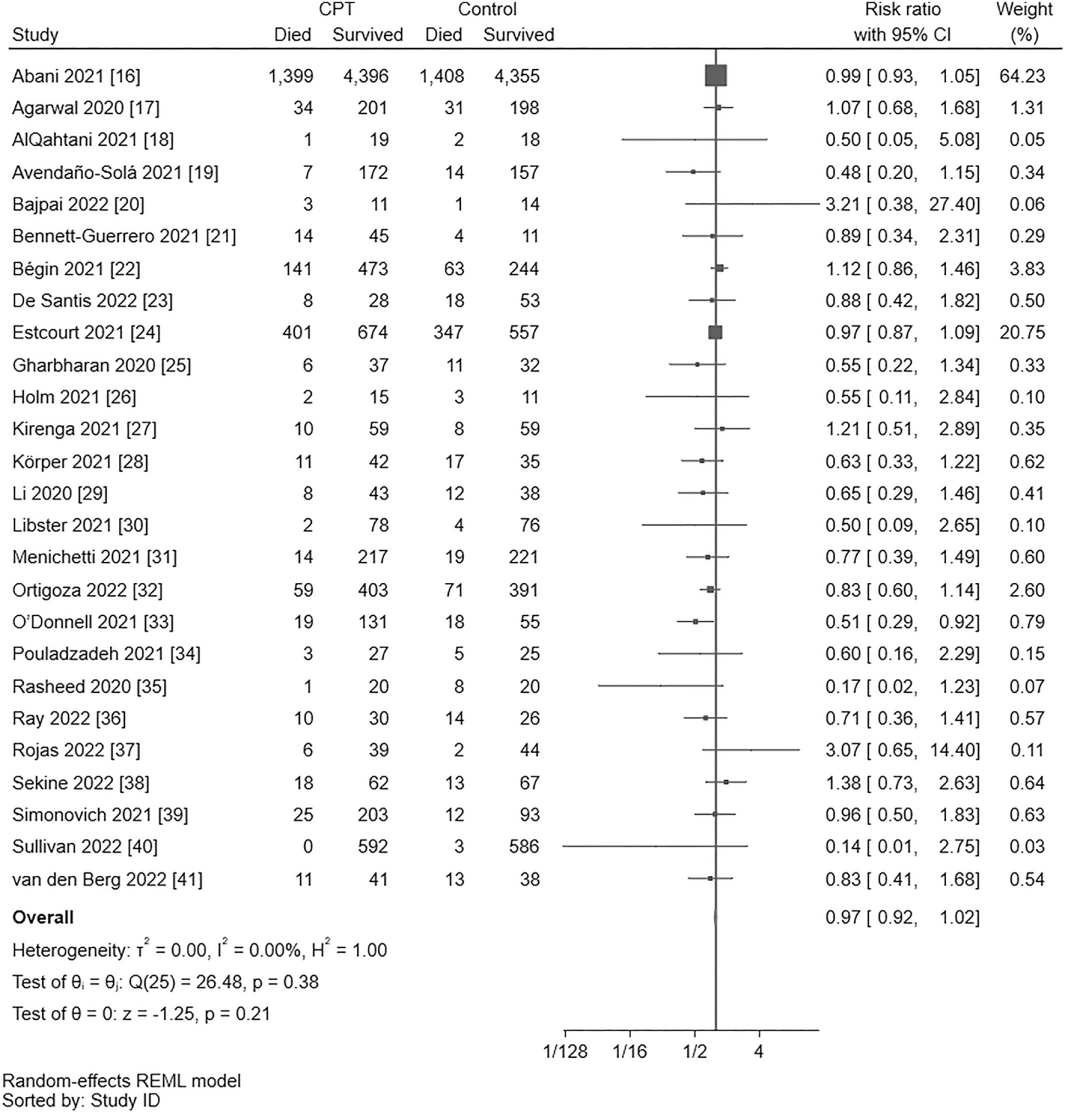
Forest plot for mortality. There is no statistically significant difference between convalescent plasma transfusion and control (risk ratio = 0.97, 95% CI = 0.92–1.02). Heterogeneity across trials is unimportant (Q(25) = 26.48, *p* = .38, *I*^*2*^ = 0.00%). *95% CI*, 95% confidence interval; *CPT*, convalescent plasma transfusion; *DF* degrees of freedom, *θ* estimated parameter, *θi* parameter of *i*th study, *θj* parameter of *j*th study, *H*^*2*^ H-squared statistic, *I*^*2*^ I-squared statistic, *p* *p* value, *Q* Cochran Q statistic, *REML* restricted maximum likelihood, *τ*^*2*^ tau-squared statistic

Twenty-Six RCT involving 19,816 patients were included in meta-analysis for mortality [16–41]. Pooling of all 26 trials showed no statistically significant effect of CPT (RR = 0.97, 95% CI = 0.92–1.02) with unimportant heterogeneity across trials (Q(25) = 26.48, *p* = 0.38, *I*^*2*^ = 0.00%) (Fig. 2).

#### Leave-one-out analysis

The results of leave-one-out meta-analysis are shown in Online Resource 4. Two studies [16, 24] seemed to be influential. Omission of the former study [16] yielded an RR of 0.90 (95% CI = 0.80–1.01), while omission of the latter [24] returned an RR of 0.90 (95% CI = 0.80–1.02). Omission of a third trial [22] was less influential yielding an RR of 0.94 (95% CI = 0.87–1.01). Nonetheless, the size and direction of the overall effect remained essentially unchanged and no statistically significant effect of CPT could be demonstrated.

#### Assessment of publication bias

Begg’s test was not statistically significant (z = − 1.28, *p* = 0.22), but Egger’s test showed possible small-study effect (z = − 2.32, *p* = 0.02). Seven missing studies were imputed with trim-and-fill and the point estimate was consequently adjusted to an RR of 0.98 (95% CI = 0.93–1.03) (Online Resource 5).

#### Grading level of evidence

Although trim-and-fill imputed seven missing studies and Egger's regression test showed possible small study effect (*p* = 0.02), effect size adjusted through trim-and-fill was practically very close to the crude (unadjusted) point estimate (RR, 0.98; 95% CI, 0.93–1.03 versus RR, 0.97; 95% CI, 0.92–1.02, respectively). So, using the GRADEpro system [15], evidence was not downgraded and there was high certainty for CPT added to standard treatment having no benefit over standard treatment alone, with an estimated effect size of seven patients getting benefit per 1,000 patients treated with CPT, and 95% CI ranging from 18 patients getting benefit to four patients possibly harmed by adding CPT to standard treatment (Table 1).

**Table 1:** Level of evidence for effect of convalescent plasma transfusion on mortality and need of invasive mechanical ventilation as graded using the GRADEpro system

Certainty assessment							No of patients		Effect		Certainty	Importance
No of studies	Study design	Risk of bias	Inconsistency	Indirectness	Imprecision	Other considerations	[CPT plus StdT]	[StdT]	Relative (95% CI)	Absolute (95% CI)		
Mortality												
26	Randomised trials	Not serious	Not serious	Not serious	Not serious	None^a^	2213/10271 (21.5%)	2121/9545 (22.2%)	RR 0.97 (0.92–1.02)	7 fewer per 1,000 (from 18 fewer to 4 more)	⨁⨁⨁⨁ High	CRITICAL
Need for IMV												
17	Randomised trials	Not serious	Not serious	Not serious	Not serious	None^b^	1186/8198 (14.5%)	1147/7885 (14.5%)	RR 1.02 (0.95–1.10)	3 more per 1,000 (from 7 fewer to 15 more)	⨁⨁⨁⨁ High	CRITICAL

*95% CI*, 95% confidence interval; *CPT* convalescent plasma transfusion, *IMV* invasive mechanical ventilation, *RR* risk ratio, *StdT* standard treatment

^a^Egger’s regression test for funnel plot asymmetry showed possible small study effect (*p* =0.02), and trim-and-fill imputed seven missing studies. Effect size adjusted with trim-and-fill was practically very close to the pooled estimate. So, evidence was not downgraded

^b^Both Egger’s regression test and Begg’s correlation tests for funnel plot asymmetry showed no small study effect (*p* = 0.25 and 0.39, respectively), but trim-and-fill imputed four missing studies. Effect size adjusted with trim-and-fill was practically very close to the pooled estimate. So, evidence was not downgraded

#### Trial sequential analysis

**Fig. 3:**
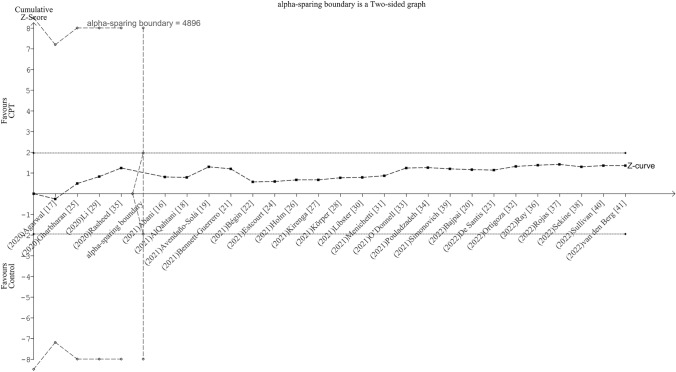
Adjusted boundaries graph for mortality obtained from trial sequential analysis. Targeting a relative risk reduction of 20% as a clinically meaningful effect size and setting the final type 1 error at .05 and type 2 error at .2, trial sequential analysis showed that the attained information size did exceed the required size. The Z-curve transected the upper futility bound (inner wedges) with inclusion of the fifth study indicating that convalescent plasma transfusion added to standard treatment is not superior to standard treatment only. *CPT* convalescent plasma transfusion

Targeting an RRR of 20% as a clinically meaningful effect size, TSA showed that the attained information (19,816 patients) did exceed the required size (4,896 patients). Besides, the Z-curve transected the upper futility bound (inner wedges) with inclusion of the fifth study [16] indicating that CPT added to standard treatment is not superior to standard treatment only (Fig. 3). Likewise, the penalized Z-curve remained well below the upper significance bounds up to inclusion of the last trial [41] (Online Resource 6).

### Need of IMV

#### Pooling of estimates and assessment of heterogeneity

**Fig. 4:**
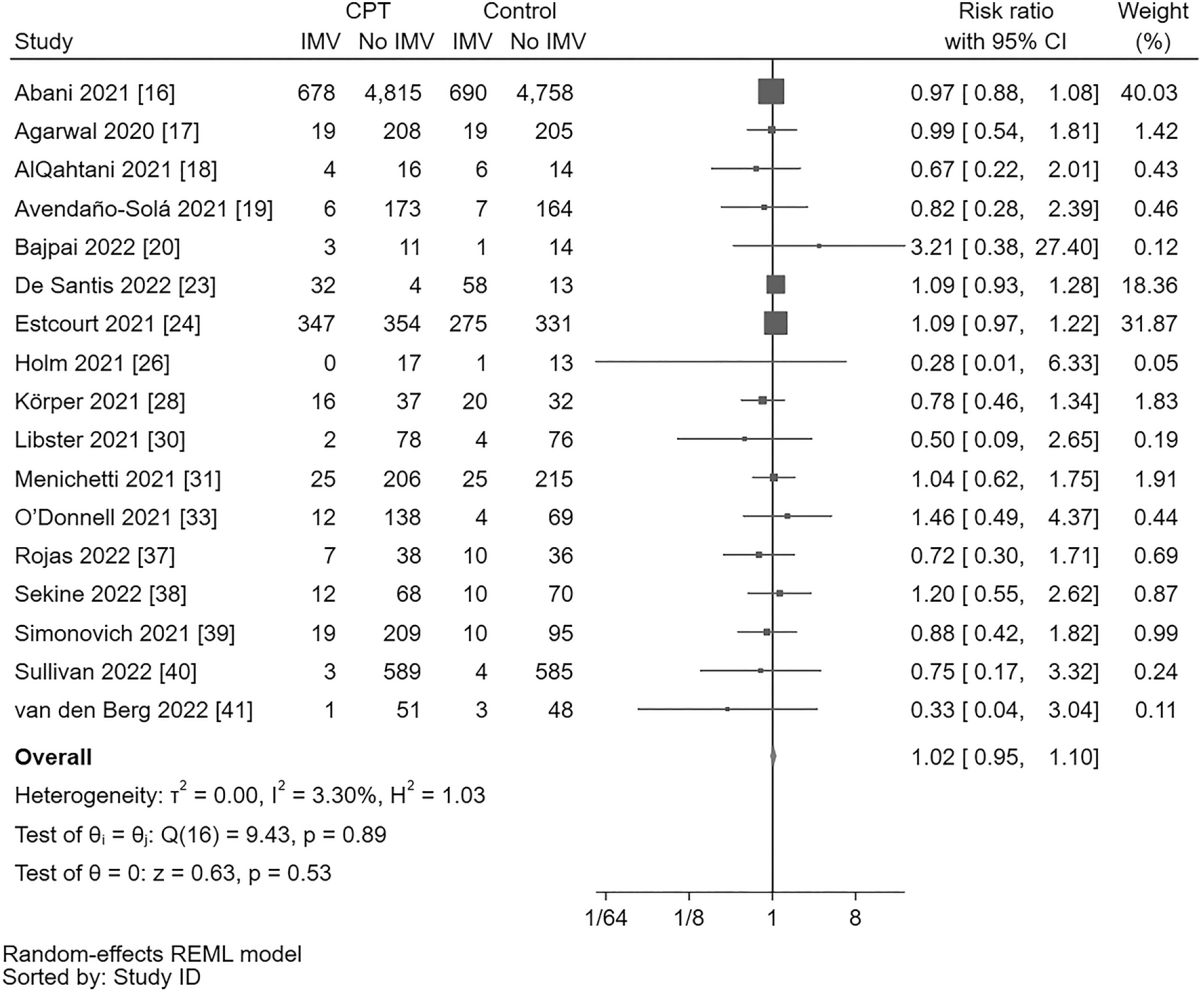
Forest plot for need of invasive mechanical ventilation. There is no statistically significant difference between convalescent plasma transfusion and control (risk ratio = 1.02, 95% CI = 0.95–1.10). Heterogeneity across trials is unimportant (Q(16) = 9.43, *p* = .89, *I*^*2*^ = 3.30%). *95% CI*, 95% confidence interval; *CPT*, convalescent plasma transfusion; *DF*, degrees of freedom, *θ* estimated parameter, *θi*, parameter of *i*th study, *θj* parameter of *j*th study, *H*^*2*^ H-squared statistic, *I*^*2*^ I-squared statistic, *p*, p value, *Q* Cochran Q statistic, *REML* restricted maximum likelihood, *τ*^*2*^, tau-squared statistic

Seventeen RCT involving 16,083 patients were included in meta-analysis for need of IMV [16–20, 23, 24, 26, 28, 30, 31, 33, 37–41]. Pooling of all 17 trials showed no statistically significant benefit of CPT (RR = 1.02, 95% CI = 0.95–1.10) with unimportant heterogeneity across studies (Q(16) = 9.43, *p* = 0.89, *I*^*2*^ = 3.30%) (Fig. 4).

#### Leave-one-out analysis

Two studies [16, 24] seemed to be influential. With the former study [16] omitted, the RR increased to 1.06 (95% CI = 0.97–1.16), while omission of the latter [24] steered the effect size to the opposite direction (RR = 0.99, 95% CI = 0.92–1.07). Omission of the study by De Santis and coworkers [23] had a lesser impact, changing the effect size to an RR of 1.01 (95% CI = 0.92–1.10). However, no statistically significant effect of CPT could be demonstrated when any of the trials was removed and, with the exception of the study by Estcourt and coworkers [24], the size and direction of the overall effect remained virtually the same (Online Resource 7).

#### Assessment of publication bias

Both Egger’s test and Begg’s test showed no small-study effect (z = − 1.14, *p* = 0.25 and z = − 0.95, *p* = 0.39, respectively). Nevertheless, trim-and-fill imputed four missing studies and the point estimate was adjusted to an RR of 1.027 (95% CI = 0.96–1.10) (Online Resource 8).

#### Grading level of evidence

Although trim-and-fill imputed four missing studies, adjusted effect size was practically very close to the crude (unadjusted) point estimate (RR = 1.03, 95% CI = 0.96–1.10 versus RR = 1.02, 95% CI = 0.95–1.10, respectively). So, using the GRADEpro system [15], evidence was not downgraded and there was high certainty for CPT added to standard treatment having no benefit over standard treatment alone, with an estimated effect size of three patients possibly harmed per 1,000 patients receiving CPT, and 95% CI limits ranging from seven patients getting benefit to 15 patients possibly harmed by adding CPT to standard treatment (Table 1).

#### Trial sequential analysis and information size

**Fig. 5:**
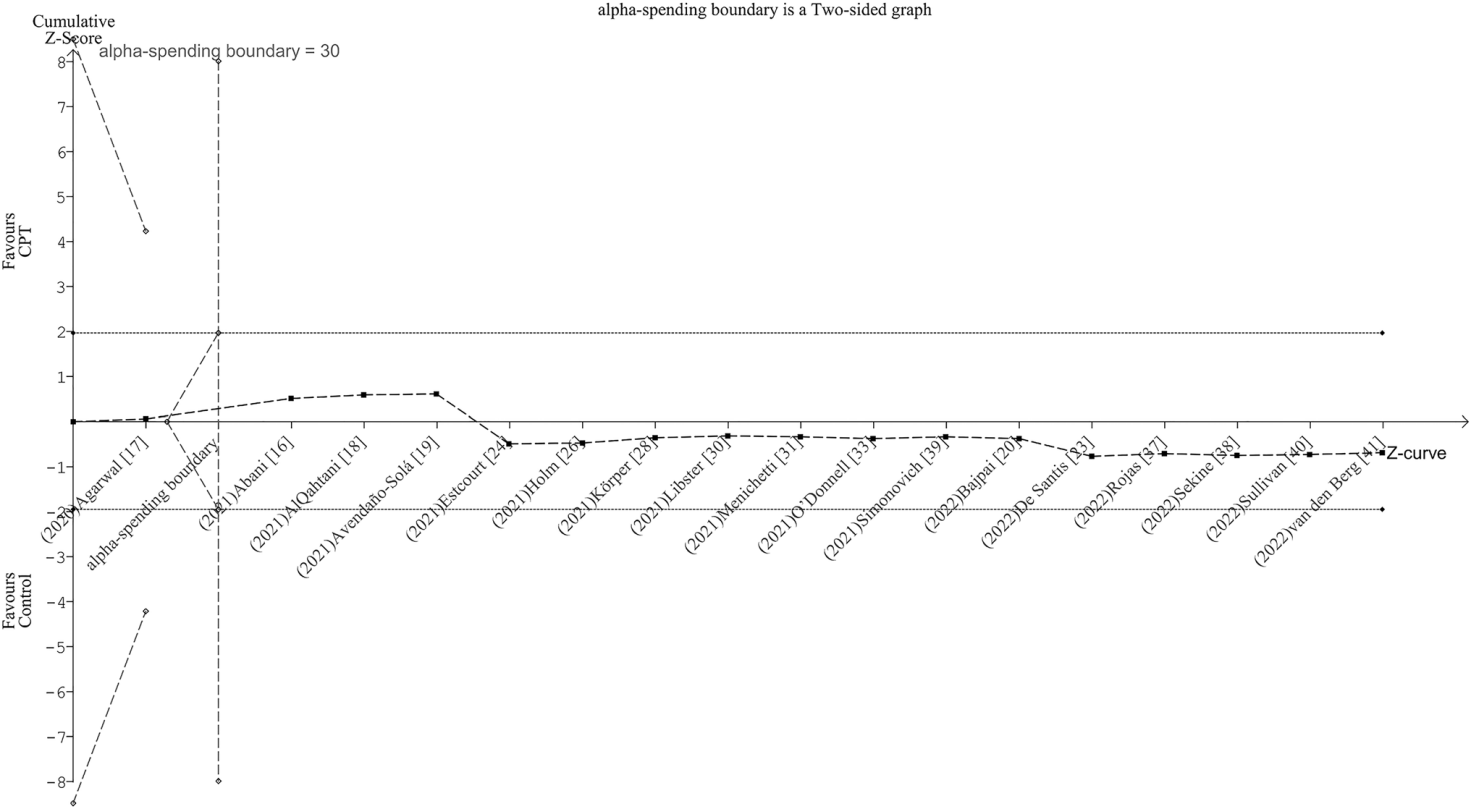
Adjusted boundaries graph for need of invasive mechanical ventilation obtained from trial sequential analysis. Targeting a relative risk reduction of 20% as a clinically meaningful effect size and setting the final type 1 error at .05 and type 2 error at .2, trial sequential analysis showed that required information size was exceeded with inclusion of the second study. The futility bounds (inner wedges) were crossed with inclusion of the second trial implying no benefit of convalescent plasma transfusion if added to standard treatment. *CPT* convalescent plasma transfusion

Targeting an RRR of 20%, TSA showed that required information size was exceeded with inclusion of the second study [16]. The futility bounds (inner wedges) were crossed with inclusion of the second trial [16], implying no benefit of CPT if added to standard treatment (Fig. 5). The penalized Z-curve strayed above and then below the null Z-value but remained within the non-significance bounds up until the last trial [41] was added (Online Resource 9).

### Transfusion-related adverse events

Twenty-three studies that included 19,475 patients did report 196 transfusion-related AE in 10,098 patients who actually received CPT [16–27, 29, 31–35, 37–41], with an overall incidence rate of approximately two events per 100 patients receiving CPT. Out of these 23 studies, four trials reported that no transfusion-related AE were observed [18, 23, 25, 34]. A considerable proportion of reported transfusion-related AE (78/196 events, 39.8%) were minor reactions such as skin rash, redness, itching, fever, pain at site of injection or urticarial rash. Serious AE accounted for 60.2% (118/196) of all reported events. Anaphylactoid reactions were responsible for 7.6% (9/118) of serious AE [16, 17, 24, 40], while dyspnea and/or desaturation [16, 17, 19, 31], TRALI [22, 40], and volume overload [22, 33] accounted for 18.6% (22/118), 1.7% (2/118) and 2.5% (3/118) of all serious AE, respectively. The remaining serious AE (82/118, 69.5%) were reported by two trials [38, 40] as Grade 3 or Grade 4 AE according to the Common Terminology Criteria for Adverse Events, version 5.0 [42].

## Discussion

Convalescent plasma transfusion has been advocated as a promising therapy for COVID-19. The results of clinical trials and reviews, however, have been inconsistent, and usually inconclusive. The present review showed that information is currently adequate to draw out clear conclusions with acceptably high certainty regarding the benefit or futility of CPT for patients with COVID-19. *Firstly*, the present evidence suggests that adding CPT to standard treatment is not associated with reduced mortality or need for IMV compared with standard treatment alone. *Secondly*, certainty for lack of benefit of adding CPT to standard treatment is high. *Thirdly*, cumulated information size has been large enough and is opting for futility of CPT. Consequently, further conduct of RCT to seek a possible benefit of CPT may not be justified.

CP has been previously utilized for management of other coronavirus-induced infections such as severe acute respiratory syndrome (SARS) and Middle East respiratory syndrome (MERS). The rationale underlying use of CP in these settings is to chelate the culprit viruses by neutralizing antibodies present in high titer in plasma of subjects who have just recovered from a recent coronavirus infection [5]. Use of CPT has been extended to the setting of COVID-19 with rather inconsistent conclusions [6–8, 43].

The present meta-analysis updates the results of a previous meta-analysis published over one year ago [9]. According to the Cochrane guidelines, the need to update a meta-analysis is determined by two fundamental considerations, whether the health problem is still relevant, and whether a sufficient number of new studies have been made available [44, 45]. The latter issue is particularly pertinent since the primary aim of a meta-analysis is to provide an answer to the question of interest as obtained from best available evidence. As a significantly large size of new information is made available, previously obtained evidence, though once valid, may become invalidated. From this perspective, the whole process is basically a function of how much new information has accumulated and how much the problem is still thriving rather than how long has elapsed since a meta-analysis was made available to readers [44, 45]. Since the review of Ling and coworkers was published [9], eight more eligible RCT including 3,044 patients have been added to the published database [23, 26, 27, 31, 32, 37, 40, 41]. This fact, combined with ongoing reports from authentic organizations about resurgence of new cases of COVID-19 and the global underestimation of the real incidence of such new cases [1], has compelled updating our evidence on this issue.

Although the present review corroborates the findings of that of Ling and colleagues [9], it has got some notable merits. Besides the more robust information size of 26 studies encompassing approximately 20,000 patients, the present review has targeted a larger effect size corresponding to an RRR of 20%. Based on an assumed mortality rate of 20% in patients receiving standard of care (SOC) only, this is translated to an absolute reduction in mortality of 4%, a clinically more relevant effect size compared with the rather meager RRR of 10% targeted by previous meta-analysts which is equivalent to an absolute reduction in mortality of as small as 2% by CPT [9]. The implication of this contrast is that the demonstrated futility of CPT by the present meta-analysis is even more decisive in view of the improved chances to demonstrate a benefit to CPT, if any, which has been achieved by the enhanced power of the analysis as a function of the larger information size as well as the bigger target effect size that is more clinically meaningful.

To the present authors’ knowledge, the meta-analysis of Ling and coworkers [9] has been preceded by four previous meta-analyses that provided inconsistent conclusions [6–8, 43]. In one meta-analysis including RCT and NRSI, the authors concluded that their results favored the efficacy of CP as a therapeutic for COVID-19 [6]. However, a deliberate examination of their results may warrant some reconsideration of these conclusions. The authors of that review reported that pooling of NRSI alone showed clear benefit of CPT on mortality while RCT showed no statistically significant benefit. The authors conducted sensitivity analysis after they excluded a large RCT [17] accounting for over one-third of statistical weight that had a directionally different effect size. This elimination redirected the effect size toward favoring CPT over SOC alone. The pretext for excluding that study from the analysis was the low level of SARS-CoV-2 antibodies in approximately two-thirds of patients in the interventional arm. However, that exclusion may have been inappropriate for the following reasons. *Firstly*, the authors of the excluded trial did conduct a modified intention-to-treat analysis where they compared the outcomes of patients receiving CPT with non-detectable neutralizing antibody titers, detectable neutralizing antibody titers or neutralizing antibody titers of 1/80 or higher *versus* controls receiving SOC only and found no statistically significant differences among the four subgroups [17]. *Secondly*, most trials investigating the effect of CP have not provided adequate information regarding the antibody titers in donated CP. *Thirdly*, there is little consensus on what should be considered as the minimum titer of SARS-CoV-2 antibodies in this clinical context. Thus, taking into consideration the potential bias induced by exclusion of an influential RCT together with reliance on evidence from NRSI, the conclusions of that meta-analysis would be questionable. Similarly, the meta-analysis by Kloypan and colleagues [8] showed that the benefit of CPT varied with the study design, the statistically significant benefit for mortality observed by pooling clinical and observational trials together being downgraded to just a trend for better outcome when only RCT or double-blinded RCT were analyzed. By the same token, an earlier meta-analysis [7] found low-certainty non-conclusive evidence from eight RCT, and low-certainty evidence from 13 cohort studies for reduction in mortality at 28 days. Another meta-analysis by Bansal and coworkers [43] provided somewhat similar results; pooling all studies showed a benefit of CPT on reducing mortality, and CPT was still beneficial when prospective or retrospective studies were pooled separately. Nonetheless, pooling RCT separately showed no statistically significant reduction in mortality. Interestingly, those authors emulated the same methodology of previous reviewers [6] and conducted sensitivity analysis excluding the study of Agarwal and colleagues [17].

The present review has got some strengths. Besides the considerably larger information size than previously published meta-analyses both in terms of number of patients and in terms of number of events, there has been little possibility of methodological bias in included RCT, the vast majority of which (22 out of 26 trials) fulfilled all five criteria of the RoB 2 Tool [11] and were qualified as at low risk of bias. *Secondly*, the present authors made use of the GRADE system [15] to determine objectively the level of certainty in obtained evidence. Inclusion of high-quality RCT exhibiting evident consistency and precision of estimated effect sizes has enhanced the level of certainty in the present meta-analysis and allowed the authors to grade the evidence as high. *Thirdly*, the present meta-analysis has made use of TSA [46] and has targeted more practical parameters for this type of analysis in order to draw empirically meaningful conclusions.

On the other hand, the present meta-analysis has got some limitations. *Firstly*, we included only studies published in English. Therefore, it is likely that a large number of relevant studies have been trimmed off by this restrictive criterion. *Secondly*, we limited our review to studying only two outcomes, mortality and need of IMV. Although most trials were concerned with other measures of clinical or biochemical improvement, the present authors found most of these outcome measures difficult to define or to quantify objectively from available data. Besides, from a clinician’s viewpoint, this couple of outcomes is almost unanimously held as the most decisive measure of success or failure of COVID-19 management. *Thirdly*, for understandable reasons, studies included in the present meta-analysis did not adopt a uniform time-frame for assessment of their main end-points of interest. So, while some authors limited their interest to in-hospital mortality [27, 30, 35], others extended surveillance to as long as 90 days from inclusion [24]. A related issue is the evident diversity among trials regarding the standard treatment offered to patients. This is foreseeable in view of the inevitable time lag that had to elapse between emergence of the pandemic and accumulation of enough information that enabled epidemiologists and clinicians to formulate justifiable frameworks for patient management. Besides, developed guidelines have been subject to a dynamic process of continuous reappraisal and update as newer pieces of information are being made available. This applies to internationally [47] as well as to regionally developed guidelines [48]. For instance, the National Institute of Health (NIH) issued the first practice guidelines for COVID-19 in April 2020. Since then, the guidelines have undergone over 60 updates pragmatically translating newer evidence into clinical practice. Though missing from the original guidelines, the NIH added a statement for corticosteroids in the June 2020 updates, then added statements for CPT and ivermectin in the September 2020 and January 2021 updates, respectively [48]. This incessant tuning up of recommendations and practice guidelines has certainly contributed to the diversity in what investigators had to offer to their patients as standard of care. Likewise, studies varied widely regarding the severity of disease at inclusion which ranged from just mild disease [30, 40] to critical illness [24, 35]. Most of the studies, however, included patients with either severe [18–21, 23, 28, 29, 32–34, 36, 37] or moderate and severe disease [22, 25, 26, 31, 41], while few studies exclusively recruited patients with moderate disease [17] or with disease of any degree of severity [16, 27]. These methodological inconsistencies may render interpretation of present results rather intricate. Nonetheless, despite this apparent variability, it is noteworthy that tests of heterogeneity did demonstrate remarkable consistency across studies as regards the estimated effect size, while sensitivity analyses did reveal practically unchanged size and direction of the overall effect as studies were sequentially eliminated. In fact, such reproducibility or consistency of effect is regarded by the GRADE system as a criterion enhancing the strength of evidence [15]. *Fourthly*, as a possible source of confounding, we detected evidence of publication bias in favor of studies displaying effectiveness of CPT in reducing mortality or need of IMV. Although this could be a reason of concern, at least theoretically, we employed the trim-and-fill method [14] to impute missing studies and adjust point estimates accordingly. In effect, difference between adjusted and unadjusted point estimates was too trivial to be of any clinical value and we opted not to downgrade the level of evidence obtained from the GRADE system [15].

## Conclusions

Availability of new information combined with resurgence of the coronavirus disease 2019 problem has impelled updating our evidence on using convalescent plasma transfusion as a possible treatment. The size of available information is adequate to conclude with high level of certainty that convalescent plasma therapy added to standard treatment of COVID-19 is not associated with reduced mortality or need of invasive mechanical ventilation compared with standard treatment alone. In view of these findings, further trials on efficacy of convalescent plasma therapy in coronavirus disease 2019 patients are probably not needed.

## Supplementary Information

Below is the link to the electronic supplementary material.

Supplementary file1 (DOCX 33 KB)

Supplementary file2 Risk of bias assessment for individual randomized controlled trials using the Cochrane Risk-of-Bias Tool for Randomized Trials Version 2.0 (RoB 2). The majority of included studies (22/26) are qualified as high-quality, fulfilling all five criteria of the RoB 2 Tool. One or more criteria of the RoB 2 Tool were either unclearly reported or completely missing in four studies, which were regarded as low-quality (TIF 4314 KB)

Supplementary file3 Overall risk of bias in randomized controlled trials as assessed using the Cochrane Risk-of-Bias Tool for Randomized Trials Version 2.0 (RoB 2) (TIF 6684 KB)

Supplementary file4 Leave-one-out forest plot for mortality. Three studies seem to be influential. Nonetheless, size and direction of overall effect remained essentially unchanged and no statistically significant effect of convalescent plasma transfusion could be demonstrated. 95% CI, 95% confidence interval; REML, restricted maximum likelihood (TIF 3719 KB)

Supplementary file5 Funnel plot for publication bias in reports of mortality. Seven missing studies (triangular markers) are imputed with trim-and-fill and effect size is negligibly adjusted to risk ratio of 0.98 (95% CI = 0.93 to 1.03). 95% CI, 95% confidence interval; θ, estimated parameter; REML, restricted maximum likelihood (TIF 2075 KB)

Supplementary file6 Penalized Z-curve from trial sequential analysis for mortality. The penalized Z-curve remained well below the upper significance bounds up to inclusion of the last trial implying that convalescent plasma transfusion added to standard treatment is not superior to standard treatment only. CPT, convalescent plasma transfusion (TIF 4027 KB)

Supplementary file7 Leave-one-out forest plot for need of invasive mechanical ventilation. Three studies seem to be influential. However, no statistically significant effect of convalescent plasma transfusion could be demonstrated when any of the trials was removed. 95% CI, 95% confidence interval; REML, restricted maximum likelihood (TIF 3332 KB)

Supplementary file8 Funnel plot for publication bias in reports of need of invasive mechanical ventilation. Four missing studies (triangular markers) are imputed with trim-and-fill and effect size is negligibly adjusted to risk ratio of 1.027 (95% CI = 0.96 to 1.10). 95% CI, 95% confidence interval; θ, estimated parameter; REML, restricted maximum likelihood (TIF 2288 KB)

Supplementary file9 Penalized Z-curve from trial sequential analysis of randomized controlled trials reporting need of invasive mechanical ventilation. The penalized Z-curve strayed above and then below the null Z-value but remained within the non-significance bounds up until the last trial was added (TIF 3421 KB)

## Data Availability

The datasets generated and/or analyzed during the current study are available from the corresponding author on reasonable request.
