# Supplementary material for: Effect of convalescent plasma transfusion on outcomes of coronavirus disease 2019: a meta-analysis with trial sequential analysis
Source: J Anesth. 2023 Feb 22;37(3):451–64. doi: 10.1007/s00540-023-03171-x (PMC9944423; doi:10.1007/s00540-023-03171-x)
Supplement: Supplementary file 1 — Supplementary file1 (DOCX 33 KB) [file 540_2023_3171_MOESM1_ESM.docx]

**Online Resource 1. Characteristics of included studies**

| Study / Year | Study design / Country | No. of participants | Age and sex* | Patients status at inclusion | CPT dose | Time of Administration | Nab titer | Std. treatment | Endpoint | Conclusion of authors |
| --- | --- | --- | --- | --- | --- | --- | --- | --- | --- | --- |
| Abani 2021 [16] | Multicenter open-label RCT / UK | Total: 11,558  CPT: 5,795  Control: 5,763 | CPT: 63·5 ± 14·7 y, 3,643 males  Control: 63·4 ± 14·6 y,  3,787 males | Mild, moderate & severe infection | 2 units 12 – 24 h apart | Median (IQR) time from symptom onset 9 (6 –12) days | Nab ≥ 1:100 | Corticosteroids, remdesivir, tocilizumab or sarilumab | Mortality, IMV | CP did not reduce mortality or need of IMV |
| Agarwal 2020 [17] | Multicenter open-label RCT / India | Total: 464  CPT: 235  Control: 229 | CPT: median (IQR) 52 (42 – 60) y, 177 males  Control: median (IQR) 52 (41 – 60) y, 177 males | Moderate infection | 2 doses 24 h apart | - | Median (IQR) Nab titer, 1:90 (1:30 – 1:240) | Antivirals, antibiotics, immunomodulators | Mortality, IMV | CP did not reduce mortality or need of IMV |
| AlQahtani 2021 [18] | Multicenter open-label RCT / Bahrain | Total: 40  CPT: 20  Control: 20 | CPT: 52.6 ± 14.9 y,  17 males Control: 50.7 ± 12.5 y, 15 males | Severe infection | 200 ml on 2 successive days | - | Mean ± SD Ab level 63.8 ± 46.8 AU/ml | Paracetamol, antivirals, tocilizumab, antibacterials | Mortality, IMV | CP did not reduce mortality or need of IMV |
| Avendano-Sola 2021 [19] | Multicenter open-label RCT / Spain | Total 350  CPT 179  Control 171 | CPT: 62.7 ± 15.7 y, 118 males  Control: 63.4 ± 14.9 y, 111 males | Severe infection | 250 –300 ml once | Median time of symptoms 6 days | Median (IQR) Nab titer, 157 (64 – 502) | HCQ, lopinavir-ritonavir, azithromycin, remdesivir, glucocorticoid, tocilizumab, anticoagulants | Mortality, IMV | CP did not reduce mortality or need of IMV |
| Bajpai 2022 [20] | Single-center open-label RCT / India | Total 29  CPT 14  Control 15 | CPT: 48.1 ± 9.1 y, 11 males  Control: 48.3 ± 10.8 y, 11 males | Severe infection | 250 ml on 2 successive days | - | Nab titer ≥ 1:80 | HCQ, azithromycin | Mortality, IMV | CP did not reduce mortality or need of IMV |
| Bennett-Guerrero 2021 [21] | Single-center double-blind RCT / USA | Total: 74  CPT: 59  Control: (standard plasma): 15 | CPT: 67 ± 15.8 y, 36 males  Control: 64 ± 17.4 y, 8 males | Severe infection | 480 ml divided into 2 doses | Median (IQR) time from symptom onset to randomization 9 (6 – 18) days | Median (IQR) Nab titer, 1:526 (1:359 – 1:786) | Glucocorticoids, remdesivir, HCQ, tocilizumab, sarilumab | Mortality | Administration of CP was not associated with reduced mortality |
| Bégin 2021 [22] | Multicenter open-label RCT / Canada, USA & Brazil | Total: 921  CPT: 614  Control: 307 | CPT: 67.7 ± 16.0 y,  362 males  Control: 67.1 ± 14.8 y,  183 males | Moderate & severe infection | 500 ml | Median time 4 days (range 2 – 7 days) | - | Antibiotics, corticosteroids, antivirals, anticoagulants | Mortality | CP did not reduce mortality or need of intubation |
| De Santis 2022 [23] | Multicenter open-label RCT / Brazil | Total: 107  CPT: 36  Control: 71 | CPT: 56.11 ± 15.15 y,  23 males  Control: 59.25 ± 12.35 y  44 males | Severe infection | 3 daily doses (600 ml each) for 3 days | 10 days from symptom onset | Minimum Nab titer 1:64 | Not mentioned | Mortality, IMV | CP did not reduce mortality or need of IMV |
| Estcourt 2021 [24] | Multicenter open-label RCT / Australia,  Canada, UK, and USA | Total: 1,979  CPT: 1,075  Control: 904 | CPT: median (IQR) 61 (52 – 69) y, 727 males  Control: median (IQR) 61 (52 – 70) y, 618 males | Critically ill patients | 2 units, mean ± SD total volume 550 ± 150 ml | within 48 h from randomization | High titer (unspecified) | Glucocorticoids, anticoagulants, antivirals, IL-6 receptor antagonists | Mortality, IMV | CP did not reduce mortality or need of IMV |
| Gharbharan 2021 [25] | Multicenter open-label RCT / Netherlands | Total: 86  CPT: 43  Control: 43 | CPT: median (IQR) 61 (56–70) y, 29 males  Control: median (IQR) 63 (55–77) y, 33 males | Moderate & severe infection | 300 ml repeated in 5 days if no improvement | Median (IQR) time from symptom onset 9 (7 – 13) days | Nab titer ≥ 1:80 | CQ, azithromycin, lopinavir/ritonavir, tocilizumab, anakinra | Mortality | CP did not reduce mortality |
| Holm 2021 [26] | Multicenter open-label RCT / Sweden | Total: 33  CPT: 17  Control; 14 | CPT: median (IQR) 80 (60–86) y, 11 males  Control: median (IQR) 65 (43–84) y, 8 males | Moderate and severe infection | 200 – 250 ml on 3 consecutive days | - | Median Nab titer  1:116 | Oxygen, betamethasone,  remdesivir,  antibiotics,  anticoagulants | Mortality, IMV | CP did not reduce mortality or need of IMV |
| Kirenga 2021 [27] | Single-center Open-label RCT / Uganda | Total 136  CPT 69  Control 67 | CPT: median (IQR) 48 (35 – 64) y, 48 males  Control: median (IQR) 53 (44 – 61) y, 49 males | Mild, moderate & severe infection | 2 units 3 h apart | - | Median (IQR) Ab level 139.5 (84.3 – 195.4) AU/ml | Corticosteroids, anticoagulants | Mortality | CP did not reduce mortality |
| Körper 2021 [28] | Multicenter open-label RCT / USA | Total: 105  CPT: 53  Control: 52 | CPT: median (IQR) 59 (53 – 65) y, 42 males  Control: median (IQR) 62 (55 –66) y, 35 males | Severe infection | 3 units (250 – 325 ml each) on day 1, 3, and 5 | Within 1 day  after randomization | Median (IQR) Nab titer 1:160 (1:80 – 1:640) | Antiviral, remdesivir, corticosteroids, tocilizumab, vasopressors, anticoagulants, antibiotics | Mortality, IMV | CP did not reduce mortality or need of IMV |
| Li 2020 [29] | Multicenter open-label RCT / China | Total: 103  CPT: 52  Control: 51 | CPT: median (IQR) 70 (62 – 80) y, 27 males  Control: median (IQR) 69 (63 – 76) y, 33 males | Severe infection | 4 – 13 ml/kg | Median (IQR) time from symptom onset 27 (22 – 39) days | IgG titer ≥ 1:640 | Antivirals, antibacterials, steroids, immunoglobulins, herbal medicines, interferon | Mortality | CP did not reduce mortality |
| Libster 2021 [30] | Multicenter Double-blind RCT / Argentina | Total: 160  CPT: 80  Control: 80 | CPT:  76.4 ± 8.7 y,  26 males  Control: 77.9 ± 8.4 y,  34 males | Mild infection | 250 ml once | < 72 h from symptom onset | IgG titer > 1:1000 | Not mentioned | Mortality, IMV | CP did not reduce mortality or need of IMV |
| Menichetti 2021 [31] | Multicenter open-label RCT / Italy | Total: 471  CPT: 231  Control: 240 | CPT: median (IQR) 65 (55 – 74) y, 150 males  Control: median (IQR) 63 (54 – 74) y, 154 males | Moderate to severe infection | 200 ml daily up to 3 infusions | - | Nab titer ˃ 1:160 | Remdesivir, glucocorticoids, LMWH | Mortality, IMV | CP did not reduce mortality or need of IMV |
| Ortigoza 2022 [32] | Multicenter Double-blind RCT / USA | Total: 924  CPT: 462  Control: 462 | CPT: median (IQR) 62 (51 – 72) y, 284 males  Control: median (IQR) 64 (54 – 74) y, 272 males | Severe infection | Single dose (250 ml) | 24 h from randomization | Median (IQR) Nab titer 1:93 (1:48 – 1:213) | HCQ, remdesivir, corticosteroids, anticoagulants | Mortality | CP did not reduce mortality |
| O’Donnell 2021 [33] | Multicenter Double-blind RCT / USA & Brazil | Total: 223  CPT: 150  Control: 73 | CPT: median (IQR) 60 (48 – 71) y, 96 males  Control: median (IQR) 63 (49 – 72) y, 51 males | Severe infection | 200 - 250 ml once | Median time from onset of symptoms 9 days | Nab titer ≥ 1:400 | Corticosteroids, remdesivir, HCQ, antibacterials | Mortality, IMV | CP reduced mortality but did not reduce need of IMV |
| Pouladzadeh 2021 [34] | Single-center single-blind RCT / Iran | Total: 60  CPT: 30  Control: 30 | CPT: 53.5 ± 10.3 y,  16 males  Control: 57.2 ± 17 y, 17 males | Severe infection | One dose (500 ml), 2nd dose if no improvement after 24 h | 4 h after admission | - | Chloroquine, lopinavir/ritonavir | Mortality | CP did not reduce mortality |
| Rasheed 2020 [35] | Multicenter RCT / Iraq | Total: 49  CPT: 21  Control: 28 | CPT: 55.66 ± 17.83 y, sex not reported  Control: 47.82 ± 15.36 y, sex not reported | Critically ill patients | 400 ml once | Mean ± SD time from onset of symptoms 14.8 ± 7.5 days | Donors with IgG index ≥ 1.25 were selected | HCQ, azithromycin, oxygen, MP | Mortality | CP did not reduce mortality |
| Ray 2020 [36] | Single-center open-label RCT / India | Total: 80  CPT: 40  Control; 40 | CPT: 30 males, age not reported  Control: 27 males, age not reported | Severe infection | 2 units on 2 consecutive days | Day of enrolment | - | HCQ, ivermectin, corticosteroids, anticoagulants, antibiotics, tocilizumab, remdesivir | Mortality | CP did not reduce mortality |
| Rojas 2022 [37] | Multicenter Single  blinded RCT / Colombia | Total: 91  CPT: 46  Control: 45 | CPT: median (IQR) 55.5 (38 –62.8) y,  33 males  Control: median (IQR) 54 (48–59) y, 31 males | Severe infection | 2 doses (250 ml each), 24 h apart | < 14 days from symptom onset, < 7 days from hospitalization | Anti-SARS-CoV-2 IgG titer ≥ 1:3200, IgA titer ≥ 1:800 | Antibiotics, corticosteroids, oxygen, anticoagulants | Mortality, IMV | CP did not reduce mortality or need of IMV |
| Sekine 2022 [38] | Single-center open-label RCT / Brazil | Total: 160  CPT: 80  Control: 80 | CPT: median (IQR) 59 (48 – 68.5) y,  49 males  Control: median (IQR) 62 (49.5 – 68) y,  44 males | Severe infection | 2 doses (300 ml each) 48 h apart | <15 days since initial  symptom onset | Median (IQR) Nab titer 1:320 (1:160 –1:960) | Glucocorticoids, immunomodulators, antibiotics, antivirals | Mortality, IMV | CP did not reduce mortality or need of IMV |
| Simonovich 2021 [39] | Multicenter double-blind RCT / Argentina | Total: 333  CPT: 228  Control: 105 | CPT: median (IQR) 62.5 (53 – 72.5) y,  161 males  Control: median (IQR) 62 (49 – 71) y, 64 males | Severe infection | Single dose of median volume of 500 ml | - | Median (IQR) total IgG Ab titer 1:3200 (1:800 – 1:3200) | Antivirals, glucocorticoids | Mortality, IMV | CP did not reduce mortality or need of IMV |
| Sullivan 2022 [40] | Multicenter double-blind RCT / USA | Total 1,181  CPT 592  Control 589 | CPT: median (IQR)  42 (32 – 54) y,  269 males  44 (33 – 55) y,  237 males | Mild infection | 250 ml once | Median (IQR) time from onset of symptoms 6 (4 – 7) days | IgG > 3.5 AU/ml at 1:101 dilution | Not mentioned | Mortality, IMV | CP did not reduce mortality or need of IMV |
| Van den Berg 2022 [41] | Multicenter double-blind RCT / South Africa | Total: 103  CPT: 52  Control: 51 | CPT: median (IQR)  54 (46 –62) y,  21 males  Control: median (IQR)  57 (47 –64) y,  21 males | Moderate to severe infection | Single dose (200 – 250 ml each) | Within 24 h from randomization | Nab titer ≥ 1:160 | Corticosteroids, anticoagulants | Mortality, IMV | CP did not reduce mortality or need of IMV |

*. Age is presented as mean ± SD unless otherwise indicated and sex as number of males

*Ab*, antibody; *AU*, antibody unit; *CP*, convalescent plasma; *CPT*, convalescent plasma transfusion; *COVID-19*, coronavirus disease 2019; *CQ*, chloroquine; *h*, hour; *HCQ*, hydroxychloroquine; *IgA*, immunoglobulin A; *IgG*, immunoglobulin G; *IL*, interleukin; *IMV*, Invasive mechanical ventilation; *IQR*, interquartile range; *kg*, kilogram; *LMWH*, low-molecular weight heparin; *ml*, milliliter; *MP*, methylprednisolone; *Nab*, neutralizing antibodies; *No*, number; *RCT*, randomized controlled trial; *SARS-CoV-2*, severe acute respiratory syndrome corona virus 2; *SD*, standard deviation; *Std*. *treatment*, standard treatment; *y*, years
