# Supplementary figures and images for: Effect of convalescent plasma transfusion on outcomes of coronavirus disease 2019: a meta-analysis with trial sequential analysis
Source: J Anesth. 2023 Feb 22;37(3):451–64. doi: 10.1007/s00540-023-03171-x (PMC9944423; doi:10.1007/s00540-023-03171-x)

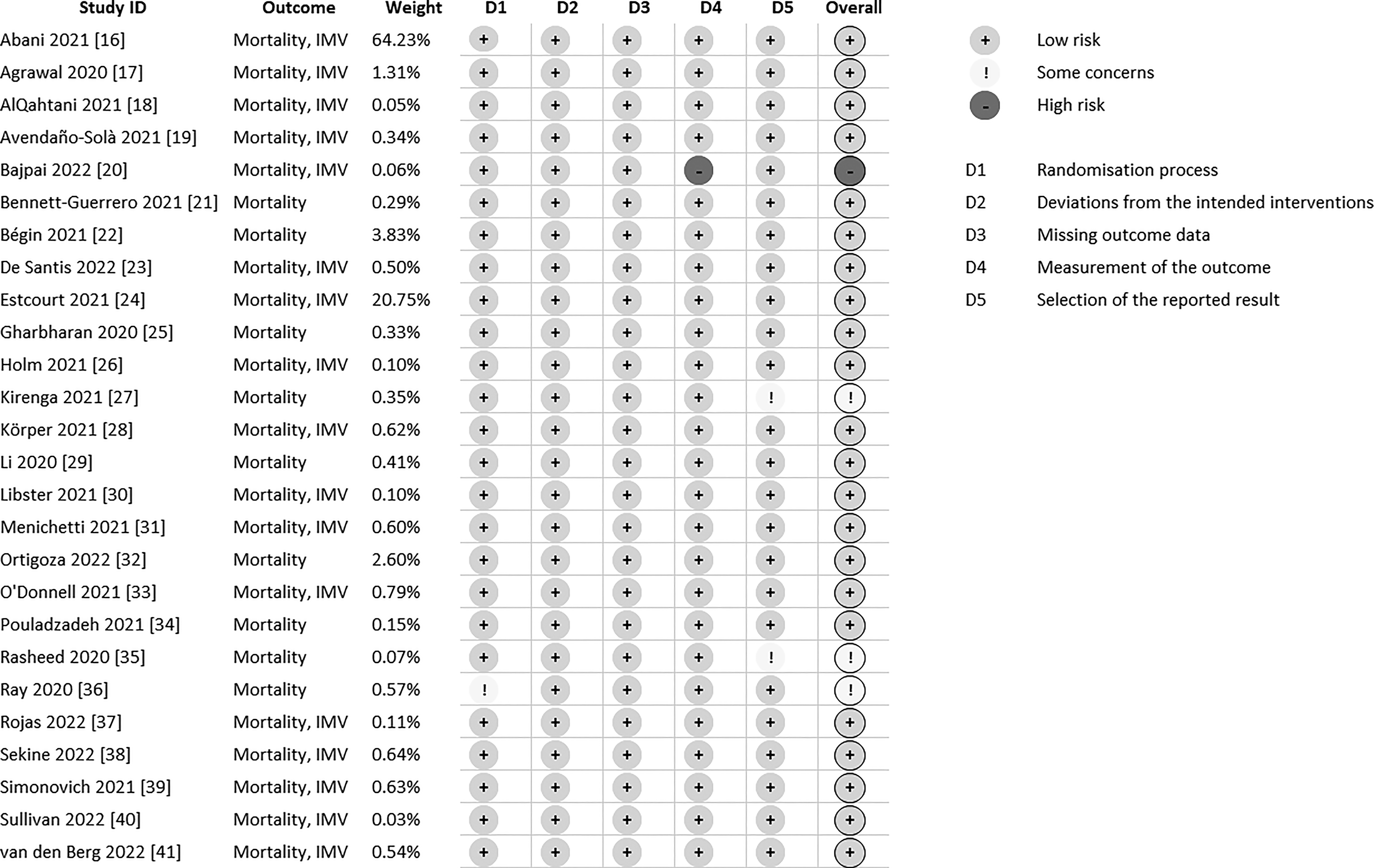

Supplement: Supplementary file 2 — Supplementary file2 Risk of bias assessment for individual randomized controlled trials using the Cochrane Risk-of-Bias Tool for Randomized Trials Version 2.0 (RoB 2). The majority of included studies (22/26) are qualified as high-quality, fulfilling all five criteria of the RoB 2 Tool. One or more criteria of the RoB 2 Tool were either unclearly reported or completely missing in four studies, which were regarded as low-quality (TIF 4314 KB) [file 540_2023_3171_MOESM2_ESM.tif]

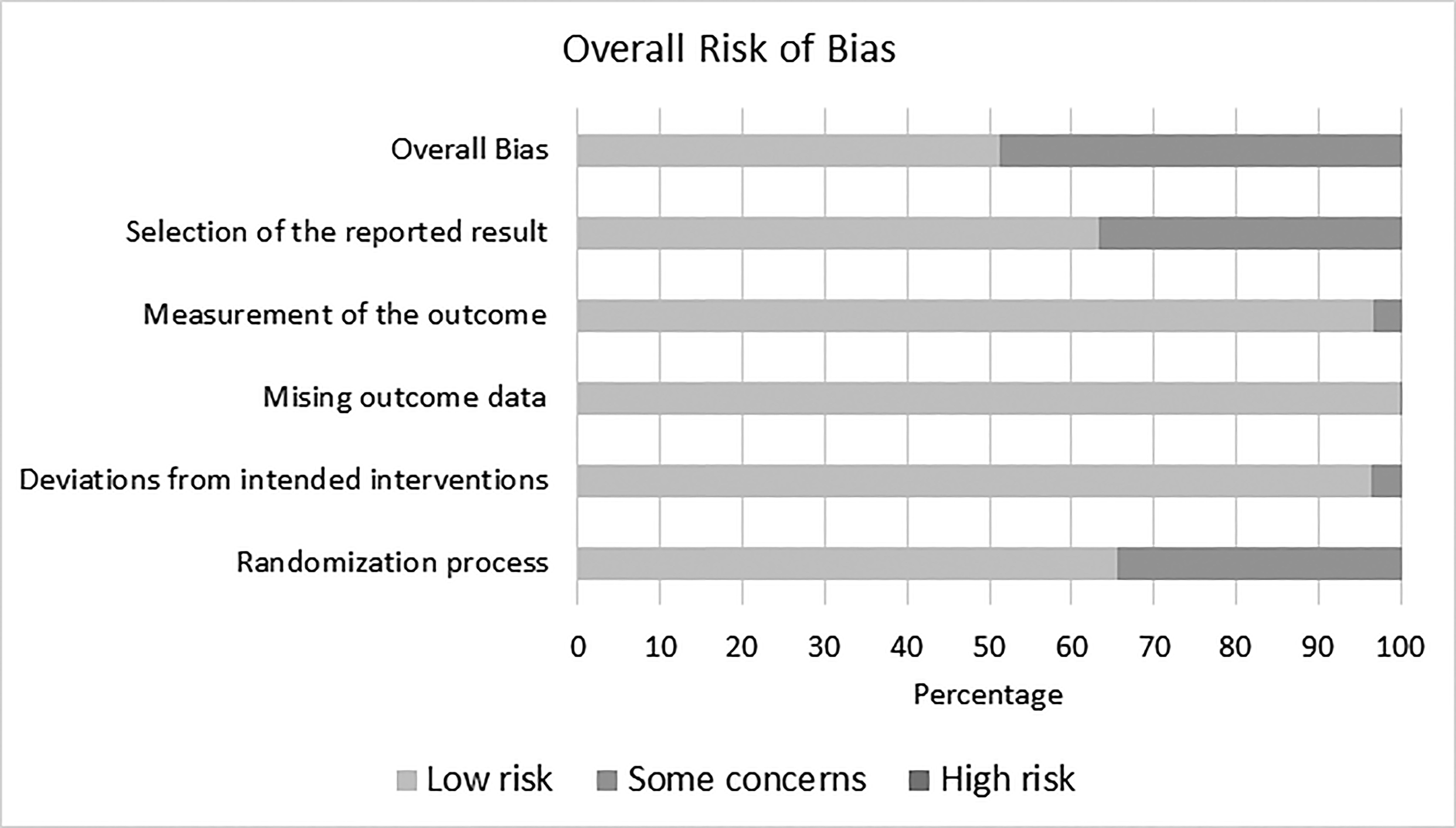

Supplement: Supplementary file 3 — Supplementary file3 Overall risk of bias in randomized controlled trials as assessed using the Cochrane Risk-of-Bias Tool for Randomized Trials Version 2.0 (RoB 2) (TIF 6684 KB) [file 540_2023_3171_MOESM3_ESM.tif]

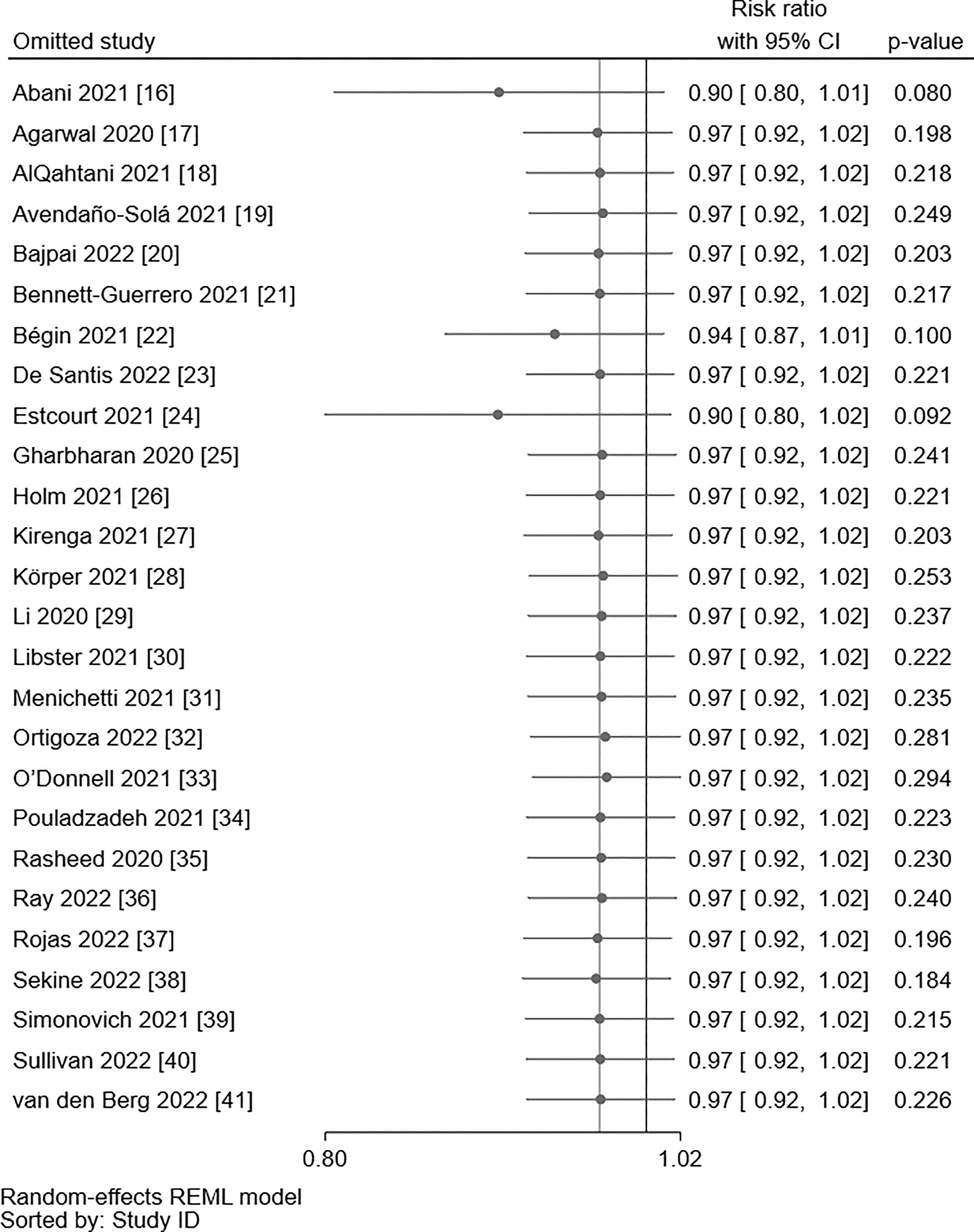

Supplement: Supplementary file 4 — Supplementary file4 Leave-one-out forest plot for mortality. Three studies seem to be influential. Nonetheless, size and direction of overall effect remained essentially unchanged and no statistically significant effect of convalescent plasma transfusion could be demonstrated. 95% CI, 95% confidence interval; REML, restricted maximum likelihood (TIF 3719 KB) [file 540_2023_3171_MOESM4_ESM.tif]

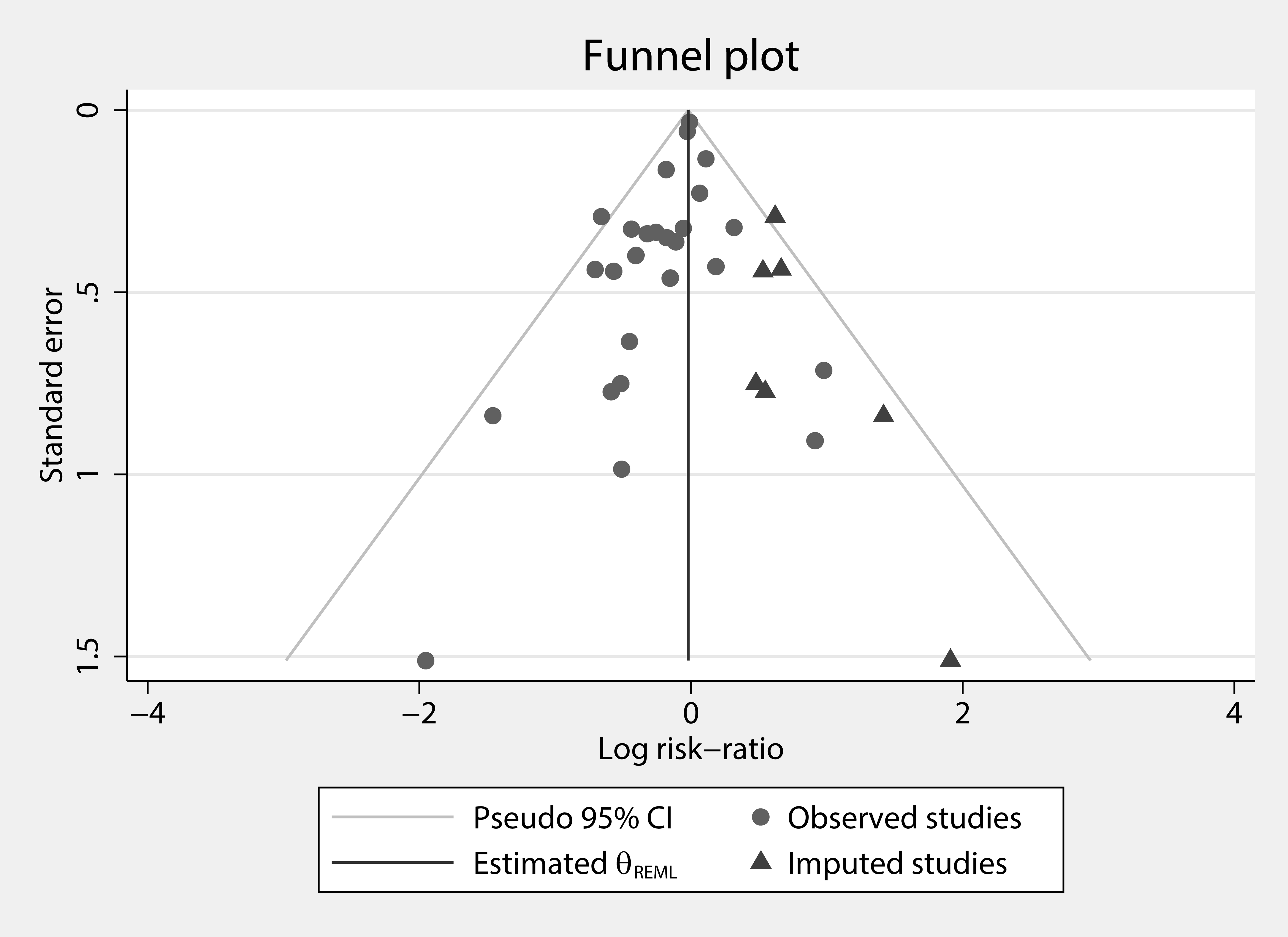

Supplement: Supplementary file 5 — Supplementary file5 Funnel plot for publication bias in reports of mortality. Seven missing studies (triangular markers) are imputed with trim-and-fill and effect size is negligibly adjusted to risk ratio of 0.98 (95% CI = 0.93 to 1.03). 95% CI, 95% confidence interval; θ, estimated parameter; REML, restricted maximum likelihood (TIF 2075 KB) [file 540_2023_3171_MOESM5_ESM.tif]

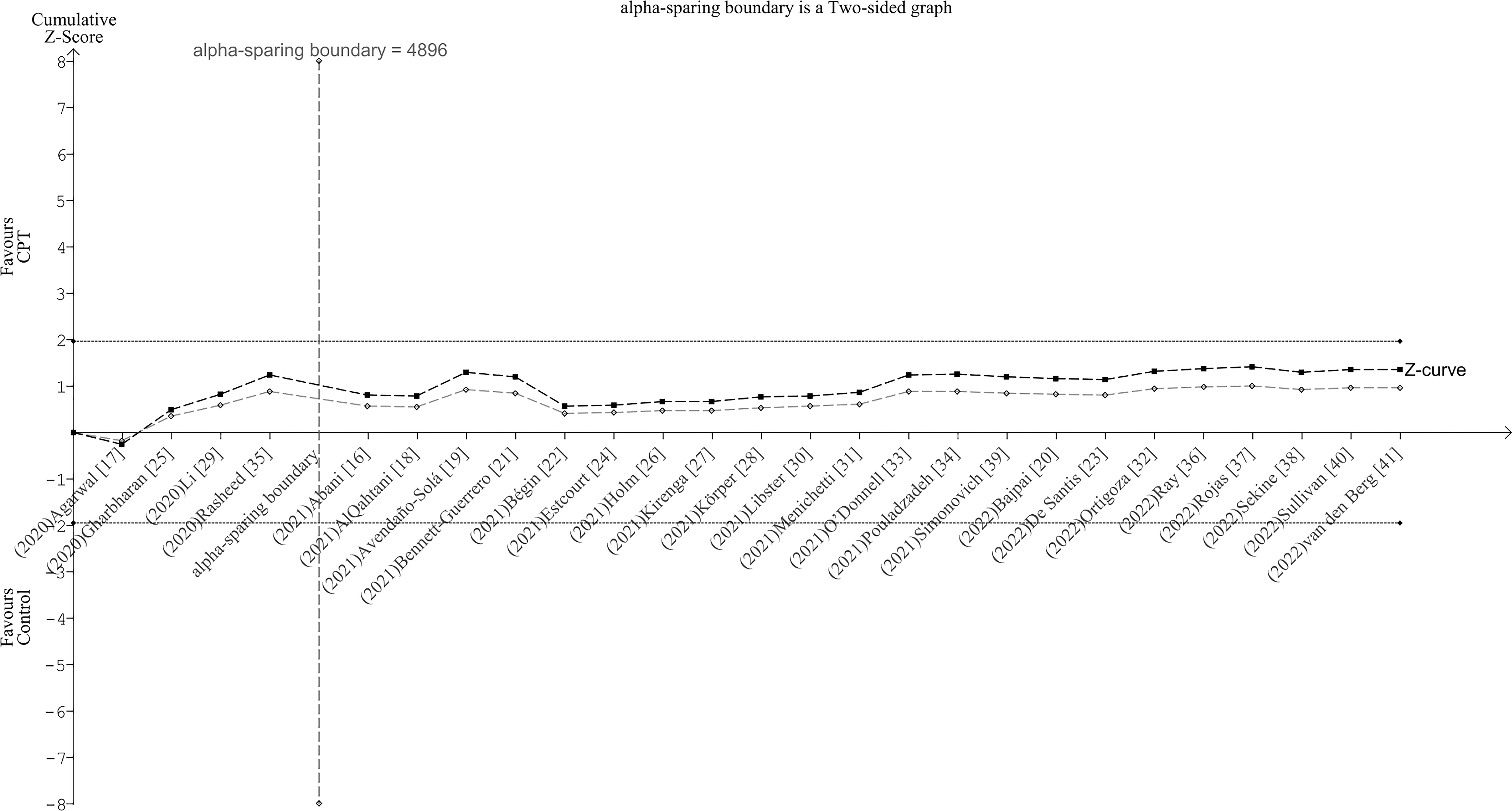

Supplement: Supplementary file 6 — Supplementary file6 Penalized Z-curve from trial sequential analysis for mortality. The penalized Z-curve remained well below the upper significance bounds up to inclusion of the last trial implying that convalescent plasma transfusion added to standard treatment is not superior to standard treatment only. CPT, convalescent plasma transfusion (TIF 4027 KB) [file 540_2023_3171_MOESM6_ESM.tif]

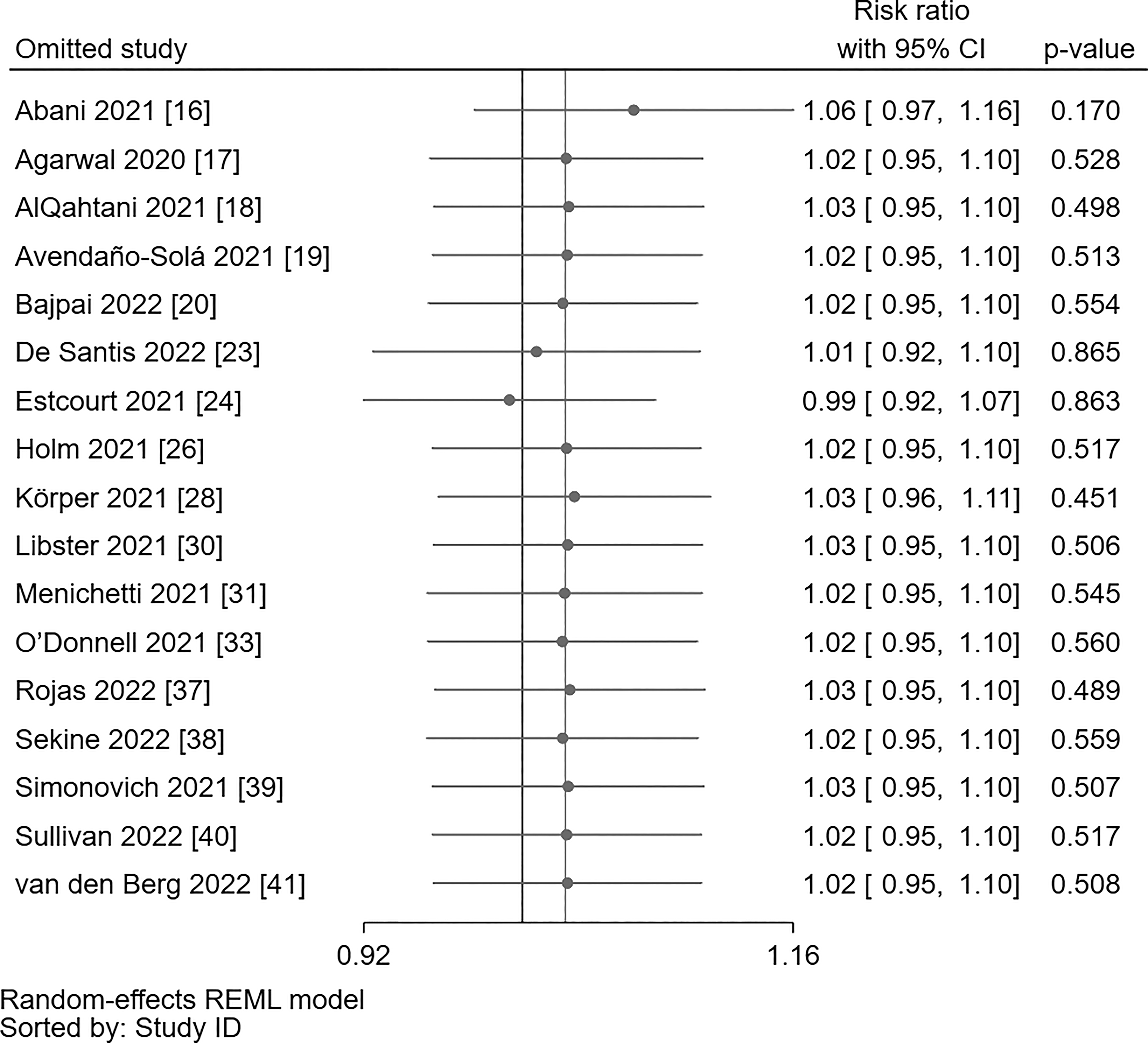

Supplement: Supplementary file 7 — Supplementary file7 Leave-one-out forest plot for need of invasive mechanical ventilation. Three studies seem to be influential. However, no statistically significant effect of convalescent plasma transfusion could be demonstrated when any of the trials was removed. 95% CI, 95% confidence interval; REML, restricted maximum likelihood (TIF 3332 KB) [file 540_2023_3171_MOESM7_ESM.tif]

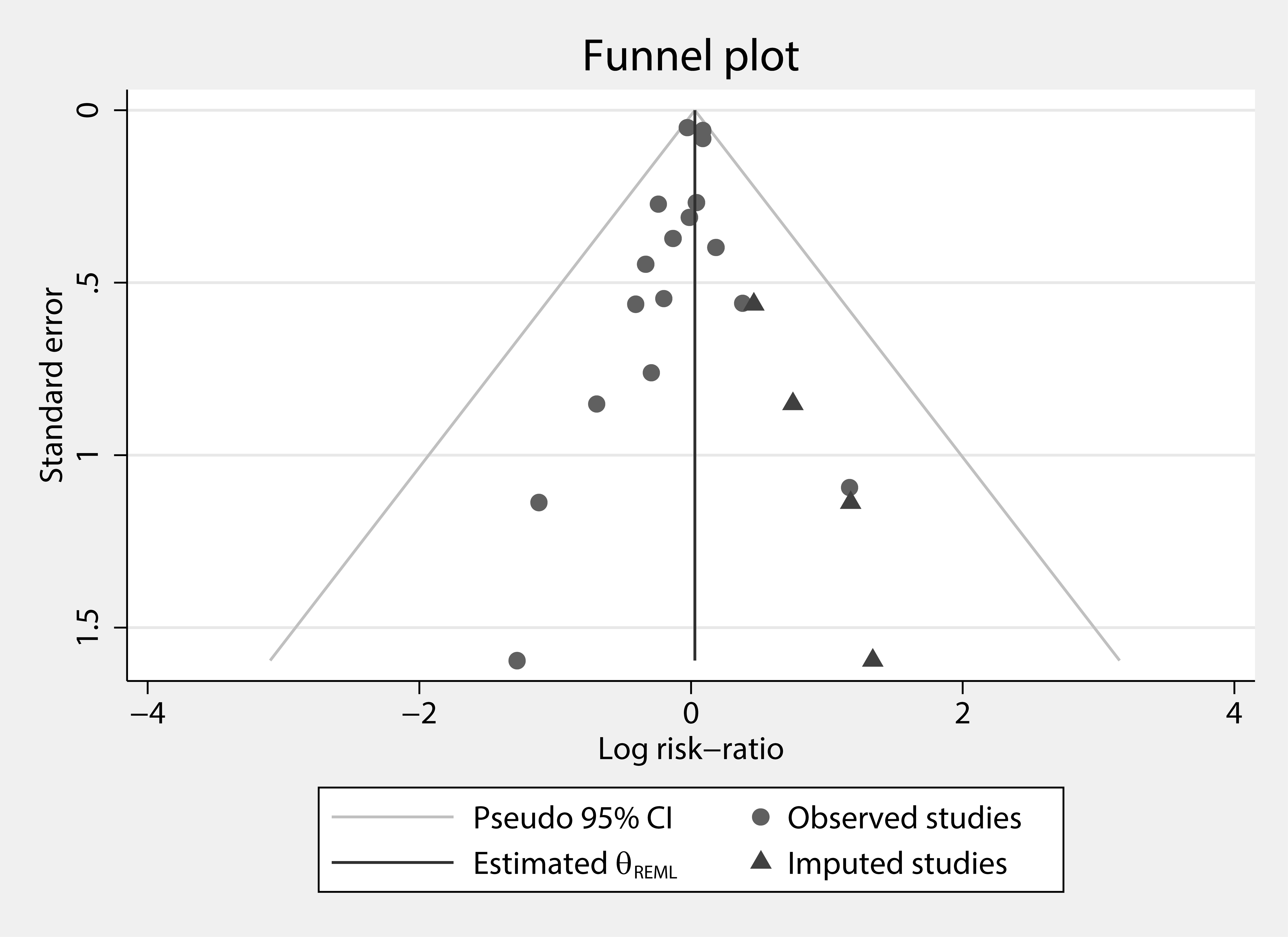

Supplement: Supplementary file 8 — Supplementary file8 Funnel plot for publication bias in reports of need of invasive mechanical ventilation. Four missing studies (triangular markers) are imputed with trim-and-fill and effect size is negligibly adjusted to risk ratio of 1.027 (95% CI = 0.96 to 1.10). 95% CI, 95% confidence interval; θ, estimated parameter; REML, restricted maximum likelihood (TIF 2288 KB) [file 540_2023_3171_MOESM8_ESM.tif]

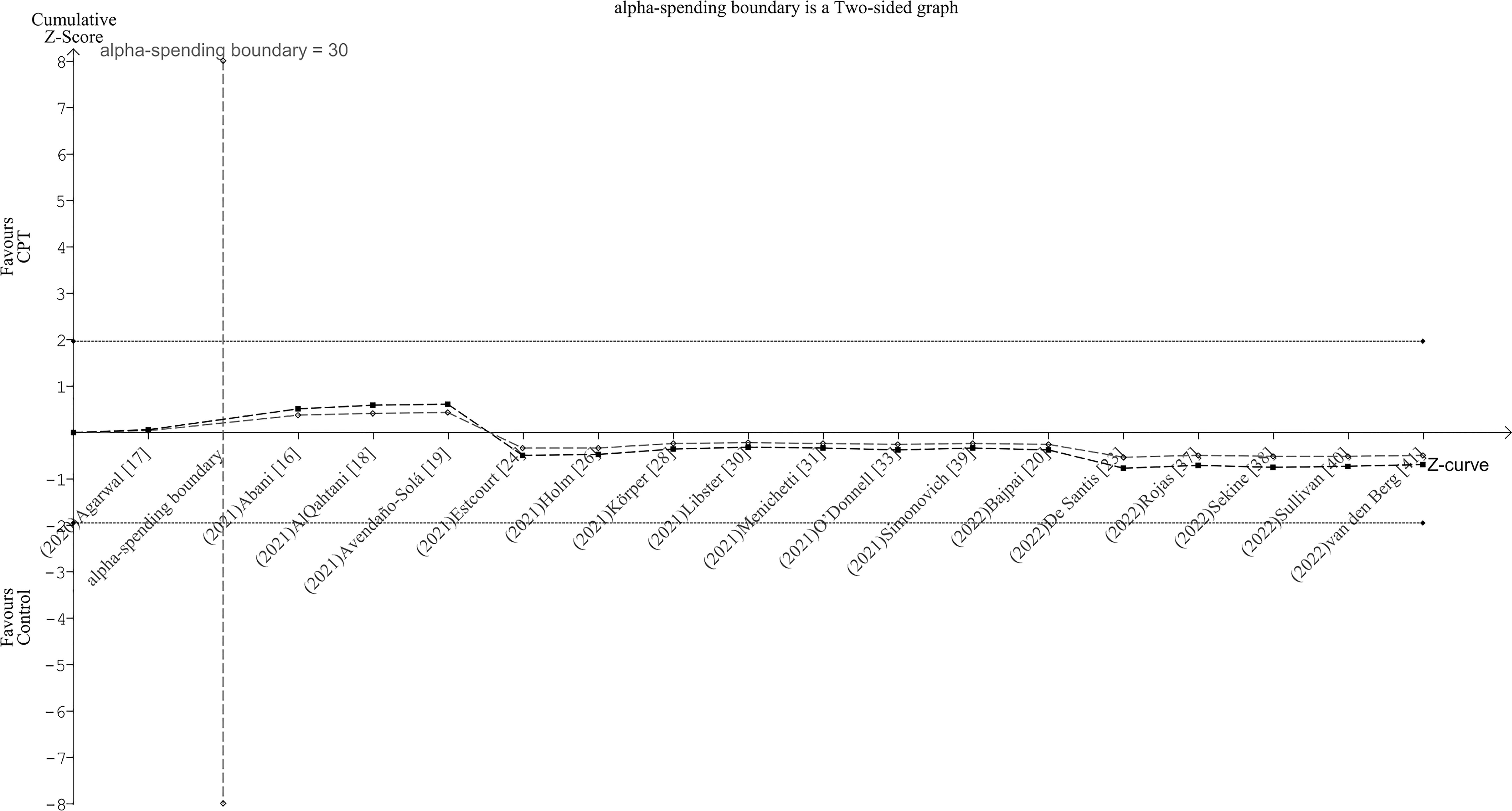

Supplement: Supplementary file 9 — Supplementary file9 Penalized Z-curve from trial sequential analysis of randomized controlled trials reporting need of invasive mechanical ventilation. The penalized Z-curve strayed above and then below the null Z-value but remained within the non-significance bounds up until the last trial was added (TIF 3421 KB) [file 540_2023_3171_MOESM9_ESM.tif]
